# The new editor—targeted genome engineering in the absence of homology-directed repair

**DOI:** 10.1038/cddiscovery.2016.42

**Published:** 2016-06-13

**Authors:** AJ Kueh, MJ Herold

**Affiliations:** 1 Walter and Eliza Hall Institute of Medical Research, Parkville, VIC 3052 Australia; 2 Department of Medical Biology, University of Melbourne, Parkville, VIC 3050, Australia

The CRISPR/Cas9 genome editing technique has become one of the most powerful tools in molecular biology. Discovered in bacteria, the minimal components required for the genome editing activity have quickly been transferred to the mammalian system; a small chimaeric RNA molecule, also called single guide RNA (sgRNA) and the Cas9 endonuclease.^1^ While the sgRNA guides Cas9 to a specific genomic locus, Cas9 induces DNA double-strand breaks (DSB) with its endonuclease activity. Through a process known as non-homologous end joining (NHEJ), imperfect repair of these DNA breaks in a gene can lead to inactivating frameshift mutations and therefore knockout of the gene.^2,3^ However, if the DSB occurs in the presence of a DNA donor template with homology to the surrounding DNA sequence, homology-directed repair (HDR) mechanisms can lead to perfect repair of the DNA and incorporation of sequences of interest. These incorporated sequences act as a medium for gene editing and can be up to several kb in length, allowing the potential correction or reversion of a disease-causing mutation. Although the CRISPR/Cas9 HDR system has the potential for clinical therapeutic interventions, there are a number of limitations and caveats with this approach, thereby restricting its widespread use in its current form.

For instance, the Cas9/sgRNA complex along with HDR DNA templates must be efficiently co-delivered into the target cells. HDR efficiency is generally low and vastly outcompeted by the error prone NHEJ pathway. To prevent DSB-induced NHEJ mutations, a number of CRISPR/Cas9 systems have been developed to minimize NHEJ while maintaining genome-editing capacity. For instance, by mutating one of the two Cas9 endonuclease domains, Cas9 nicks genomic DNA rather than causing DSB. This eliminates NHEJ mutations and drastically reduces unintended base changes at target genes.^4^ However, Cas9 nicking activity is several times less potent in stimulating HDR as compared with Cas9 DSB. Another advance in HDR-mediated repair is the choice of the DNA donor template. Recently, it was described that single strand DNA oligos with asymmetric homology sequences dramatically improve the HDR mechanism.^5^ Moreover, the authors also showed that an enzymatically inactive Cas9 is also capable of mediating HDR at very low frequencies, which might be a way forward when introducing targeted mutations but lowering off target effects. However, a drawback of these systems is that the HDR DNA template has to be present at the genomic locus at the same time as the Cas9/sgRNA complex cuts or binds the DNA; otherwise no specific targeting will occur.

An exciting new DNA-editing enzyme, which does not require a DNA template to introduce specific mutations, has been described by Komor *et al.*^6^ It was demonstrated, that a modified Cas9 fused to a cytosine deaminase mediates single base genome editing with high efficiency but without DSBs. Cytidine deaminases catalyze the deamination of cytosine (C), to yield uracil (U). Although most cytidine deaminases act on RNA, a number of them can convert C→U in single-stranded DNA as well.^7^ Following the C→U conversion, DNA replication or the DNA repair machinery can then convert the resulting U:G heteroduplex to an A:T base pair, mediating gene editing in the process.

Komor *et al.*^6^ identified rat APOBEC1 with the highest cytidine deaminase activity *in vitro*. As cytidine deamination requires single-stranded DNA for robust enzymatic activity, they proposed the fusion of APOBEC1 to the amino terminus of a Cas9 nickase complex to (a) precisely target ABOBEC1 deaminase activity to specific gene loci for gene editing, (b) allow Cas9-guided RNA–DNA ‘R-loop’ complex formation to expose single-stranded DNA for efficient deamination and (c) utilize Cas9 nickase activity to stimulate DNA mismatch repair and the permanent conversion of the U:G heteroduplex to A:T following cytidine deamination. In addition, a bacteriophage-derived uracil DNA glycosylase inhibitor (UGI) was fused to the C terminus of Cas9 nickase, inhibiting the reversion of the U:G pair back to the original C:G pair by cellular uracil DNA glycosylases.^8^ This APOBEC1–dCas9 (A840H)–UGI gene-editing complex was named as a base editor (BE).

Although this approach limits the repair to C→U conversions, there are >300 human diseases in which a conversion from C→U would be beneficial. However, one problem for specifically editing C→U is the position of the target base relative to the PAM site required for Cas9 recognition (4–8 bases upstream of the PAM with base 7 being the preferred target base). One possible solution to this is to use other Cas9 variants that have different PAM recognition sites.^9,10^ An alternative approach could involve the use of a different deaminase enzyme, which has different binding properties at the DNA, leading to the recognition of the target C at positions other than the ones described by Komor *et al.*^6^

Komor *et al.*^6^ also demonstrated the use of BE to genetically modify two disease relevant mutations *in vitro*. First, the study modified the Alzheimers’ risk factor protein APOE4 in immortalized mouse astrocytes by introducing a R158C gene editing event with up to 75% efficiency, compared with using Cas9 DSB-stimulated HDR which mediated only 0.3% gene editing at this locus. Second, the study demonstrated BE correction of the cancer-associated T163C p53 mutation in human HCC1954 breast cancer cell lines with up to 7.6% efficiency, compared with undetectable Cas9 DSB-stimulated HDR at this locus. However, these figures might be an underrepresentation as cells expressing mutant p53 are probably reliant on the expression of this mutant form. Hence, a higher editing efficiency at the mutant p53 gene may have occurred, but could no longer be detected as the gene-edited cells could have already undergone growth arrest or apoptosis.^11^ Importantly, InDels were maintained at very low levels in the BE system, with a gene correction:InDel ratio of 23, compared with 0.17 for the Cas9 HDR system.

It should be noted that the BE system is also susceptible to off-target gene editing, in a similar manner to traditional CRISPR/Cas9 systems. Furthermore, due to the 5 nucleotide window of BE deaminase activity, even on-target sites are susceptible to unintended C→U base changes, which may compromise protein-coding sequences. It will be interesting to see if future improvements to this system can narrow down the gene-editing window to a single base without compromising editing efficiency.

In summary, these findings from Komor *et al.*^6^ highlight the potential of modifying and refining CRISPR/Cas9 complexes to achieve high gene targeting efficiencies with minimal associated InDels, both of which are absolute requirements for the use of gene editing in therapeutic contexts. Furthermore, the BE system does not rely on HDR for gene editing and does not require double- or single-stranded donor DNA templates, making the system attractively simple to use for suitable gene targets. Therefore the BE system will surely be a great asset for basic researchers introducing their desired mutations *in vitro* and potentially *in vivo*. It remains an open question, whether this system will pave its way into the clinic for CRISPR/Cas9-mediated gene therapies. However, time will tell which system (Figure 1) will reliably perform with highest efficiency and lowest off-target effects.

## Figures and Tables

**Figure 1:**
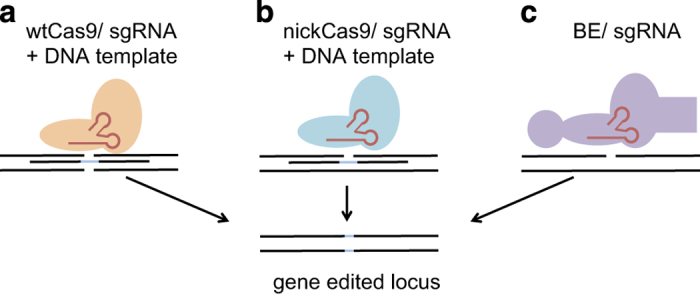
Targeted editing of DNA by three different approaches. (**a**) A complex of wild-type Cas9 and sgRNA together with a DNA template is introduced into cells. DSB lead to HDR-mediated incorporation of the desired sequence (light blue). (**b**) A complex of a nickase Cas9 and sgRNA together with a DNA template is introduced into cells. Nicking of the DNA leads to HDR-mediated incorporation of the desired sequence (light blue). (**c**) A complex of BE (APOBEC1-dCas9 (A840H)-UGI) and sgRNA leads to the deamination of a C and therefore conversion into U (T). This results in gene-editing without the need for a HDR and the use of DNA templates.
